# Green electrochemical method for the synthesis of nitro and azo derivatives based on mefenamic acid

**DOI:** 10.1038/s41598-022-05009-0

**Published:** 2022-01-20

**Authors:** Parvaneh Amooshahi, Sadegh Khazalpour, Ameneh Amani, Hossein Masoumi

**Affiliations:** 1 Faculty of Chemistry, Bu-Ali Sina University, Hamedan, 65178-38683 Iran; 2 Nahavand Higher Education Complex, Bu-Ali Sina University, Hamedan, 65178-38683 Iran

**Keywords:** Chemical biology, Electrochemistry, Green chemistry, Organic chemistry

## Abstract

Electrochemical study of mefenamic acid (**MFA**) was carried out with details in water/ethanol mixture by the various voltammetric techniques. The results showed that the oxidation of MFA is highly dependent on pH and follows the E_ir_ mechanism. The E_pA1_-pH diagram plotted based on the differential pulse voltammograms shows two linear segments, 66 and 26 mV/pH slope. Also, the diffusion coefficient and the surface excess, Ӷ* of MFA in aqueous buffered solution, determined by using the single potential-step chronoamperometry and chronocoulometry methods. Electrochemical nitration of MFA in an aqueous solution and the presence of nitrite ion (1) were both investigated by the cyclic voltammetry and controlled-potential coulometry techniques. Our results indicate that the oxidized form of MFA participates in a Michael-type addition reaction with nitrite ion (1) to form the corresponding Nitromefenamic acids (**MFA-4-NO**_2_ and **MFA-5-NO**_2_). Also, in another part, a computational study based on the density functional theory (DFT/B3LYP) was performed for the prediction of the best possible pathway in the nucleophilic addition of nitrite ion (1). The electrochemical reduction of produced nitromefenamic acids was investigated using cyclic voltammetry and controlled-potential coulometry techniques. Eventually, two new azo derivatives have been generated via electroreduction of produced nitromefenamic acids and conduction of diazotization reaction, respectively. Both nitro and azo products are approved as paints.

## Introduction

**Figure 1:**
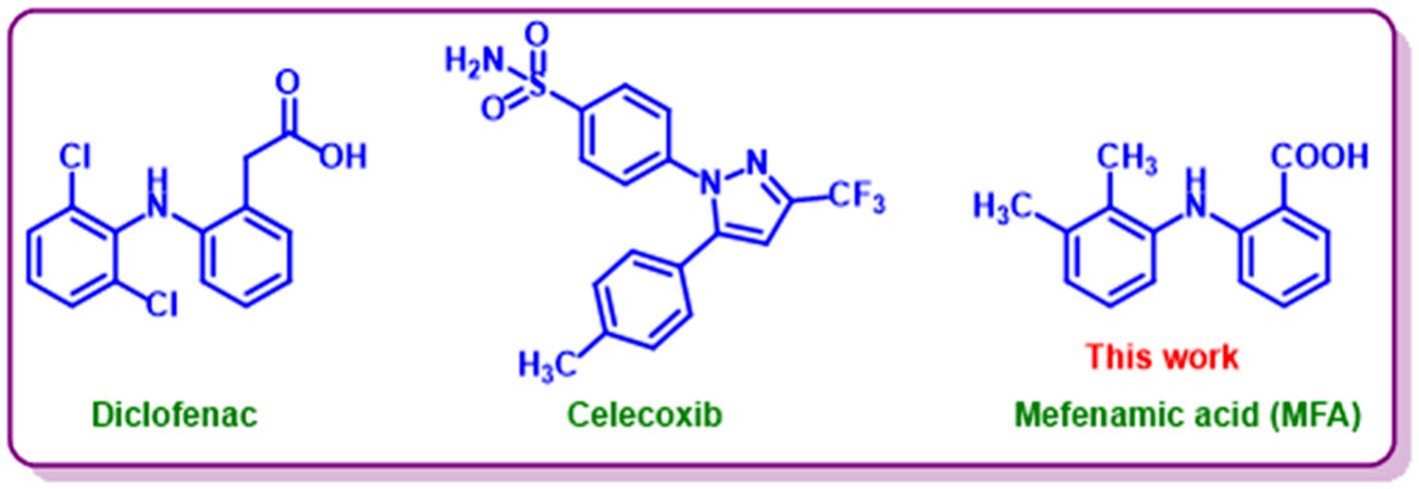
The structure of (**A**) Diclofenac, (**B**) Celecoxib, and (**C**) Mefenamic acid (**MFA**) as NSAIDs containing aromatic amines.

Amines and generally nitrogen-containing compounds are one of the most abundant organic molecules. Mefenamic acid (**MFA**) is a member of the aromatic amines (Fig. 1) with an amine group among two benzene rings. This drug is classified in the category of non-steroidal anti-inflammatory drugs (NSAIDs) as the most common and available drug^1,2^. (Fig. 1). Mefenamic acid is used widely for its anti-inflammatory, antipyretic, and analgesic effects^3,4^ and relief of pains such as dental pain, headache, due menstrual cycles pain^5,6^ and to treat several pathologies including sports injuries, menorrhagia, and primary dysmenorrhea^7,8^. Mefenamic acid overdose causes the generation of toxic metabolites, which as a result leads to bloody diarrhea, nausea, and adverse immunologic reaction in the gastrointestinal tract, kidney, and liver^6–10^. As we know, the electrochemical oxidation of aromatic amines is an intricate process that results in the production of different products, depending on the structure and electrolysis conditions^11,12^.

On the other hand, the chemical industries tend to use cleaner synthetic methods, and hence the products may have a minimum impact on the natural environment. The nitration of organic compounds is a critical industrial process^13^. The diverse applications of nitro species, especially nitro-aromatic compounds, have aroused much interest and led to extensive study of these bunch of compounds. These compounds are used in the production of nitro and azo dyes in the industry^14^, and they also can be used in the electrochemically production of polymeric azobenzenes with prominent optical characteristics^15^.

Due to the importance of these compounds, various studies were performed about the nitration methods. The nitration methods which were used can be thermal, photochemical, or electrochemical^13^. Nitration of aromatic hydrocarbons with electrochemical methods has been suggested by various authors^16,17^.

The numerous studies have been performed on the electrochemical behavior of some drugs^18–23^.

In this study, at first, the electrochemical oxidation of mefenamic acid (**MFA**) was investigated with details by the various voltammetric techniques in an aqueous solution. The diffusion coefficient and the surface excess, Ӷ* of **MFA** in aqueous buffered solution, determined by using the single potential-step chronoamperometry and chronocoulometry methods. The electrochemical nitration of **MFA** was investigated in the presence of nitrite ions via cyclic voltammetry and controlled-potential coulometry techniques in an aqueous solution. Besides, the distribution of natural charge, the relative Gibbs free energy, and also the HOMO–LUMO energy gap of the desired intermediates were investigated by the density functional theory (DFT/B3LYP) with 6–311 + G (2d, p) basis set^24,25^, to predict the best possible pathway for the nucleophilic addition of nitrite ion and also to evaluate the thermodynamic and the kinetic stability of intermediates. Afterward, the electrochemical reduction of produced nitromefenamic acids was investigated using electrochemical methods. Two new azo derivatives have been synthesized with electroreduction of produced nitromefenamic acid and performing the diazotization reaction, respectively. These products are approved as new dyes.

## Results and discussion

### Electrochemical oxidation of mefenamic acid (MFA): the effect of pH

**Figure 2:**
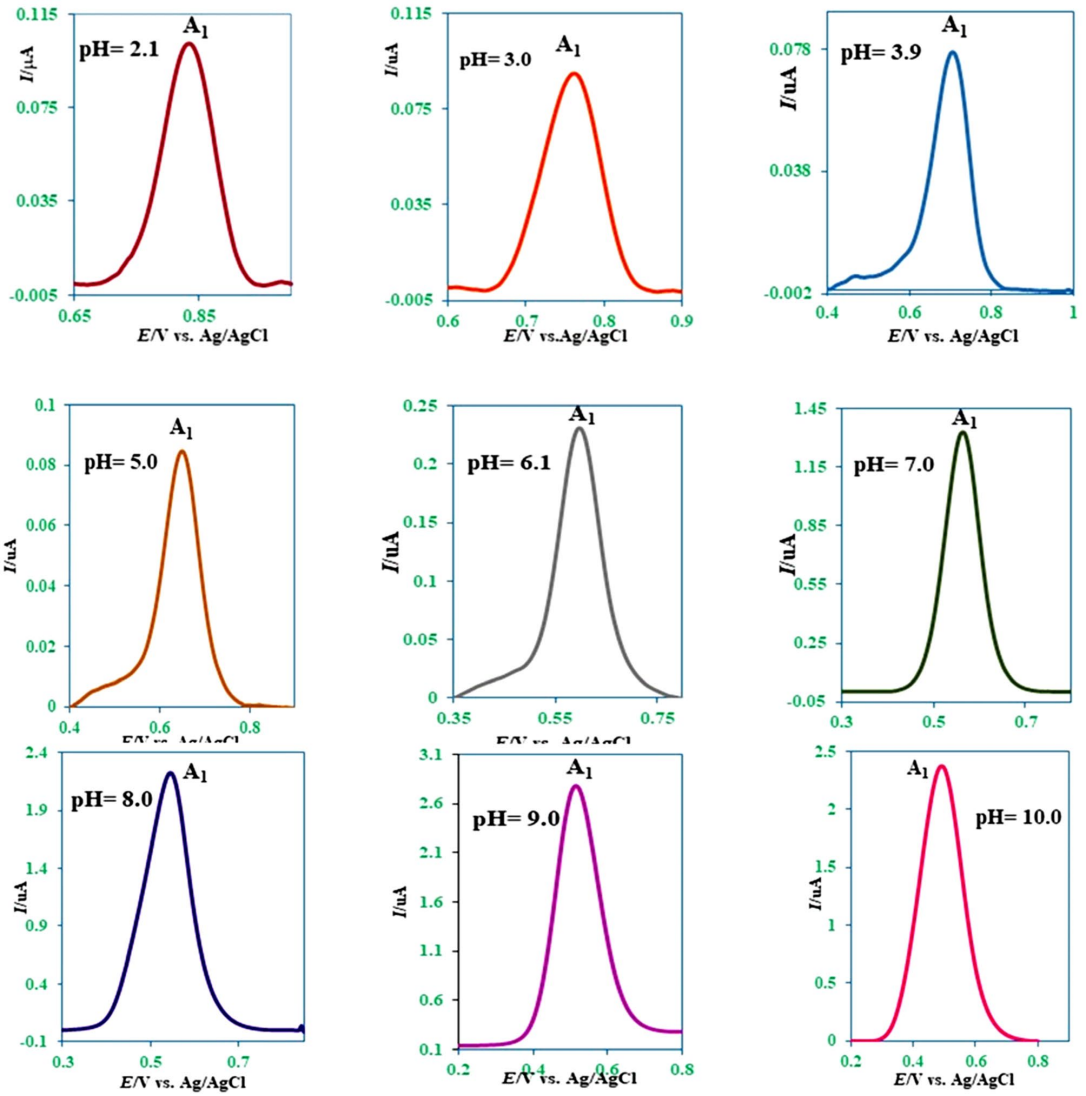
Differential pulse voltammograms of mefenamic acid (**MFA**) (0.4 mM) at the surface of glassy carbon electrode in buffered solutions with various pH_s_ and same ionic strength (c = 0.2 M). Potential scan rate: 10 mV/s. Room temperature.

The differential pulse voltammograms of the mefenamic acid (**MFA**) (0.4 mM) in buffered solutions and a variety of pHs (c = 0.2 M) are presented in Fig. 2.

**Figure 3:**
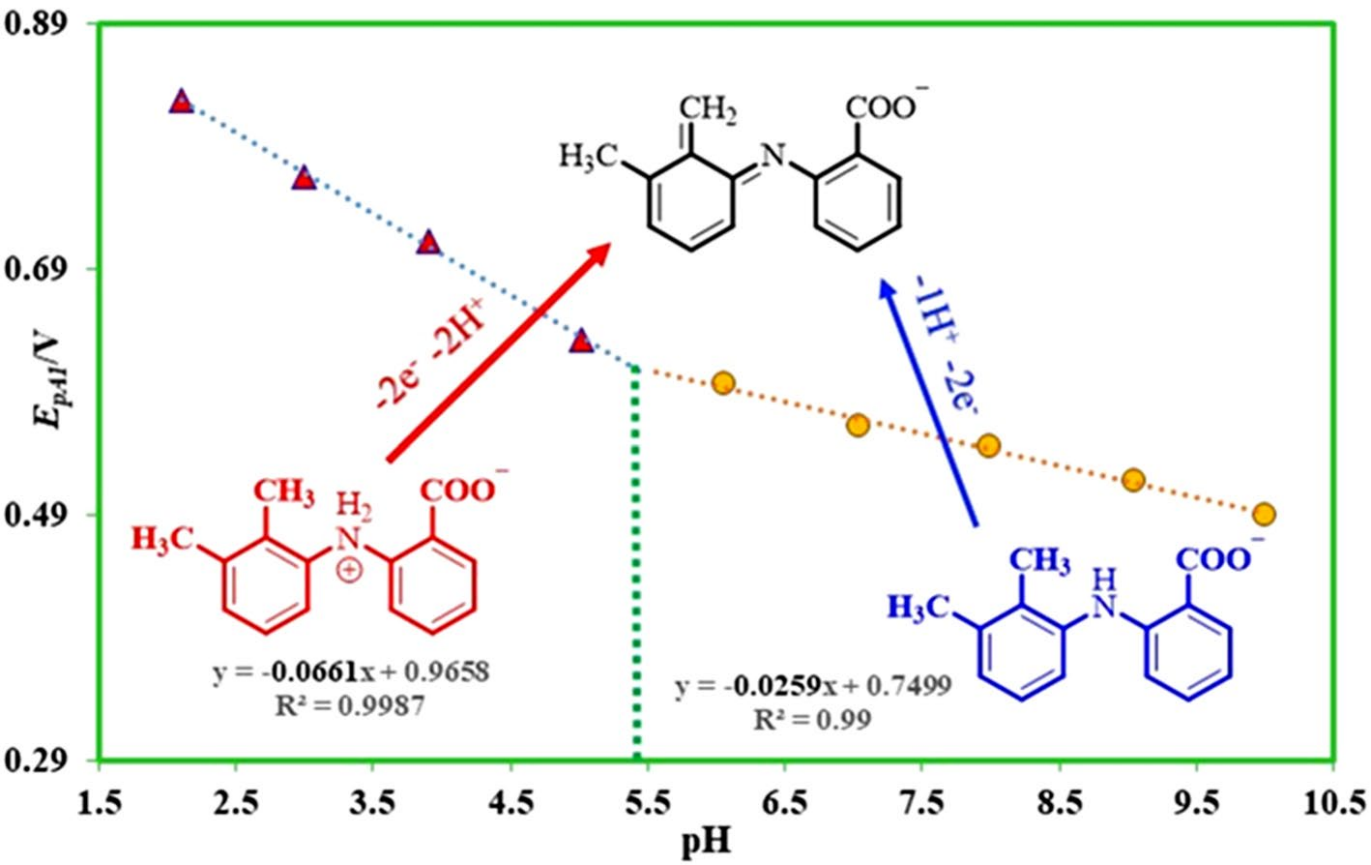
The potential-pH diagram of **MFA**.

As a predictable phenomenon, because of the participation of proton(s) in the oxidation reaction of **MFA**, by increasing pH, the peak potential for peak A_1_ (*E*_p_^A1^) shifted to the less positive potentials (Fig. 2). A potential-pH diagram is depicted in Fig. 3, via plotting the *E*_p_^A1^ against pH values. The linear regression equation is:

$$E_{{\text{p}}}^{{\prime {\text{A1}}}} = E_{{{\text{p}}\,({\text{pH}} = 0)}}^{{{\text{A1}}}} - \, \left( {{2}.{3}0{3}\,{\text{mRT}}/{\text{2F}}} \right){\text{ pH}}{.}$$

In this equation *E*_pA1(pH=0)_, is the peak potential for peak A_1_ at pH = 0.0, m is the number of protons taken apart in the reaction. R, T, and F have their general meaning. This diagram (*E*_pA1-pH_) has two linear segments with various equations and slopes around pH values 5.4.

In pH < 5.4:

$$E_{{{\text{pA1}}}} = \, 0.{96} - \, 0.0{66}\,{\text{ pH}}\,\,{\text{or}}\,\,{\text{slope}} = {66}\,{\text{mV}}/{\text{pH}}{.}$$

In pH > 5.4:

$$E_{{{\text{pA1}}}} = \, 0.{75} - 0.0{26}\,{\text{ pH}} \,\, {\text{or}} \,\, {\text{slope }} = {26}\,{\text{ mV}}/{\text{pH}}{.}$$

These findings show that three forms of **MFA** (one oxidized form, and two reduced forms) can be produced in the diffusion layer with the changing of pH and electrode potential.

**Figure 4:**
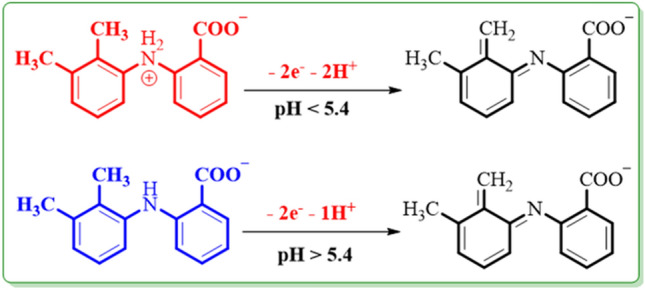
Oxidation pathways of **MFA** in different pH values.

**Figure 5:**
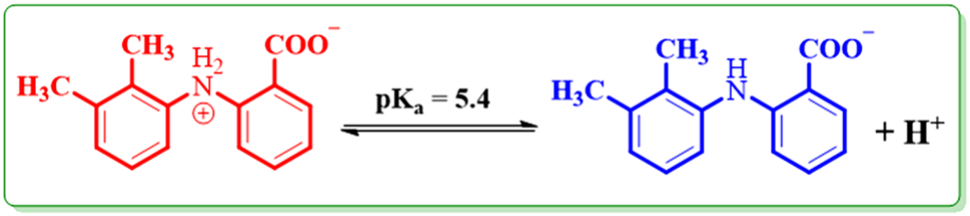
Acid/base equilibrium of **MFA**/**MFA**^−^.

According to the attained linear slopes of the linear regression equations (*E*_pA1-pH_) in Fig. 3, it can be deduced that the electrode reaction at pH < 5.4 is a two-electron/two-proton, and at pH > 5.4 is a two-electron/one-proton process. (Fig. 4). Also, the accounted pK_a_ for the **MFA/MFA**^**−**^ equilibrium is 5.4 (Fig. 5).

### Chronoamperometry method for determination of diffusion coefficient of MFA

**Figure 6:**
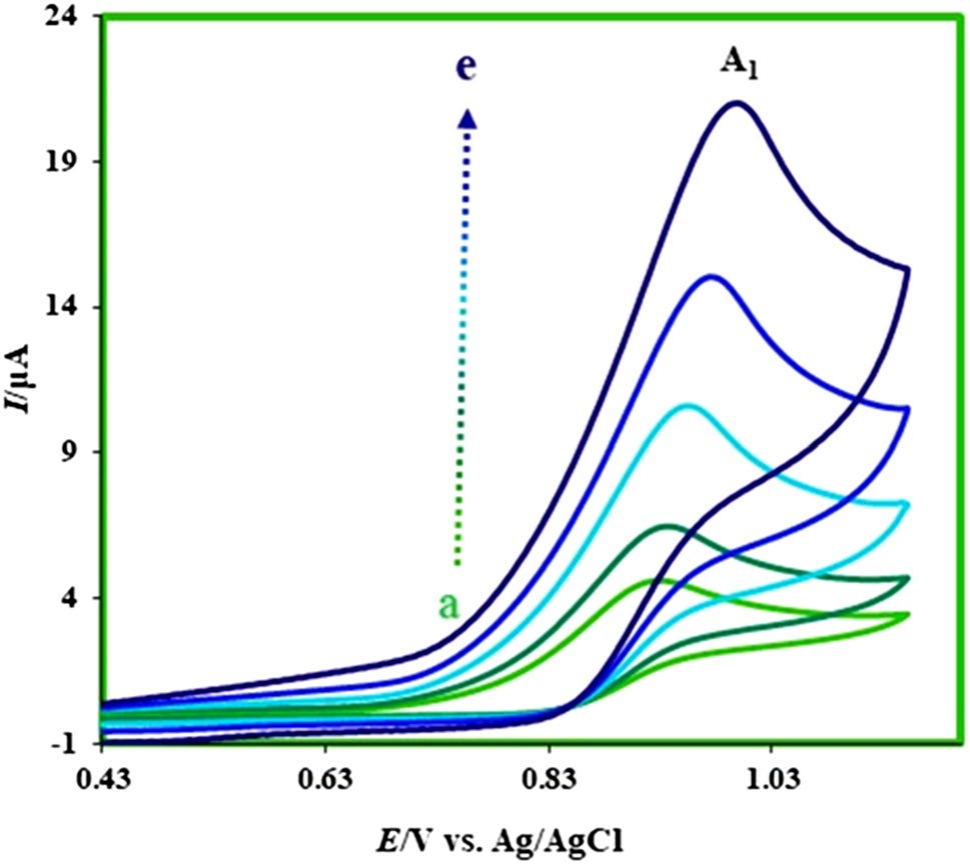
Cyclic voltammograms of **MFA** (1.0 mM) at glassy carbon electrode in ethanol containing MgClO_4_ (1.0 M) in various scan rates. Scan rates from a to e are: 5, 10, 25, 50, and 100 mV/s. Room temperature.

In this part of studies for reducing the absorption effects, the cyclic voltammograms of **MFA** (1.0 mM) were recorded in ethanol containing MgClO4 (0.1 M) and in various scan rates (Fig. 6). Based on the obtained voltammograms, the potential of 0.95 V was selected for the chronoamperometry measurements.

In continues, the chronoamperometry method is applied for the measurement of the diffusion coefficient of **MFA**. This method has been known as an essential and precise way for the determination of diffusion coefficients^26,27^ and the rate constants of homogeneous reactions^28,29^. At first *r*_*d*_ as the radius of the disk electrode should be calculated. For this reason, the cathodic scan was recorded by the linear sweep voltammetry (LSV) in a 1.0 mM solution of potassium ferricyanide.

**Figure 7:**
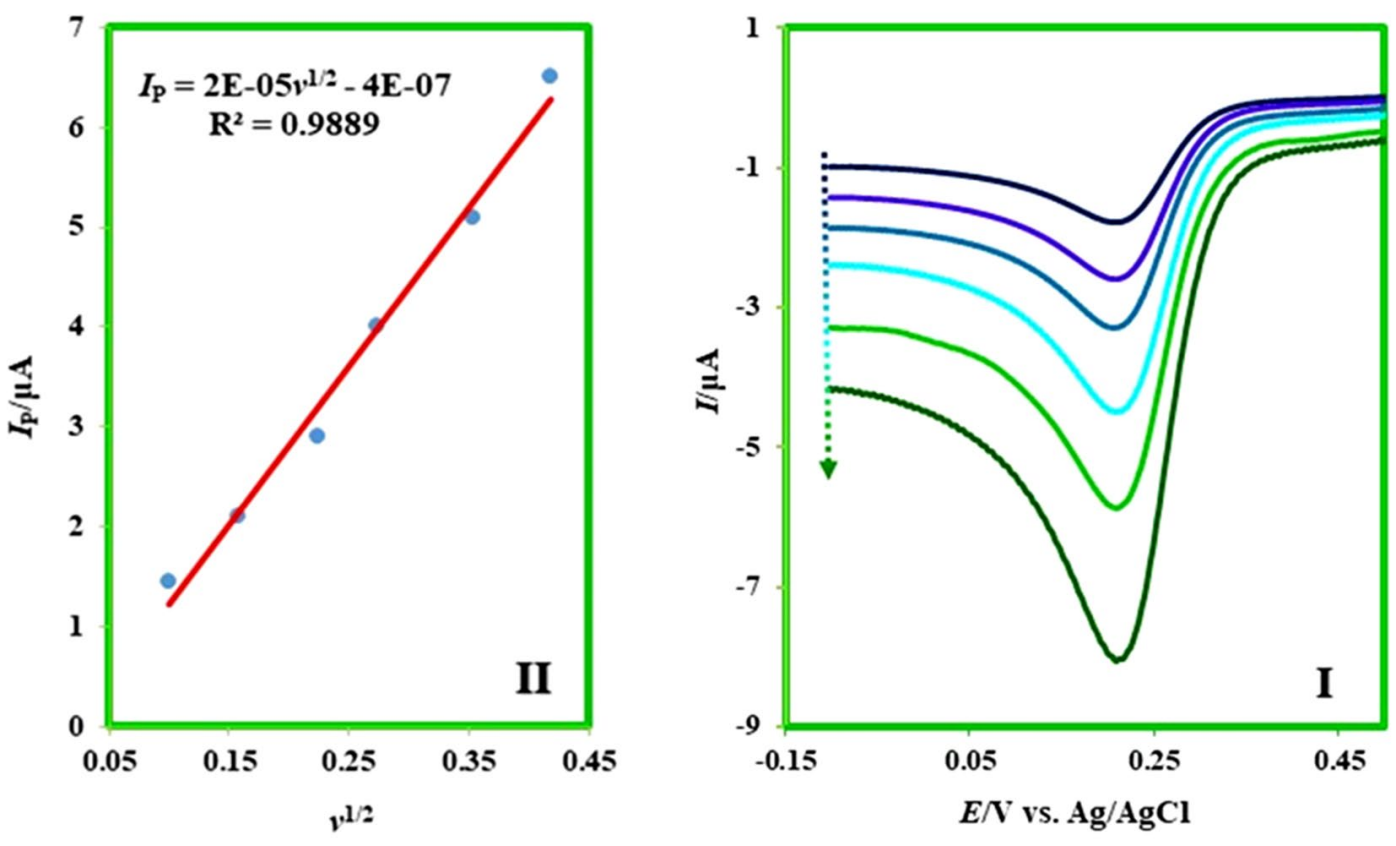
(**I**) Linear sweep voltammograms of 1.0 mM potassium ferricyanide containing KCl (1.0 M) at glassy carbon electrode in aqueous solution in various scan rates. Scan rates from a to f are: 10, 25, 50, 75, 125, and 175 mV/s. (**II**) *I* − *v*^1/2^ diagram of curves in (**I**). Room temperature.

Afterward, the radius of the electrode (equal to 0.093 cm) calculated by drawing the *I* − *v*^1/2^ and using the Randles–Sevcik equation (*I*_*p*_ = 2.69 × 10^5^ × *A* × *n*^3/2^ × *D*^1/2^ × *C*_0_ × *υ*^1/2^) (Fig. 7).

**Figure 8.**
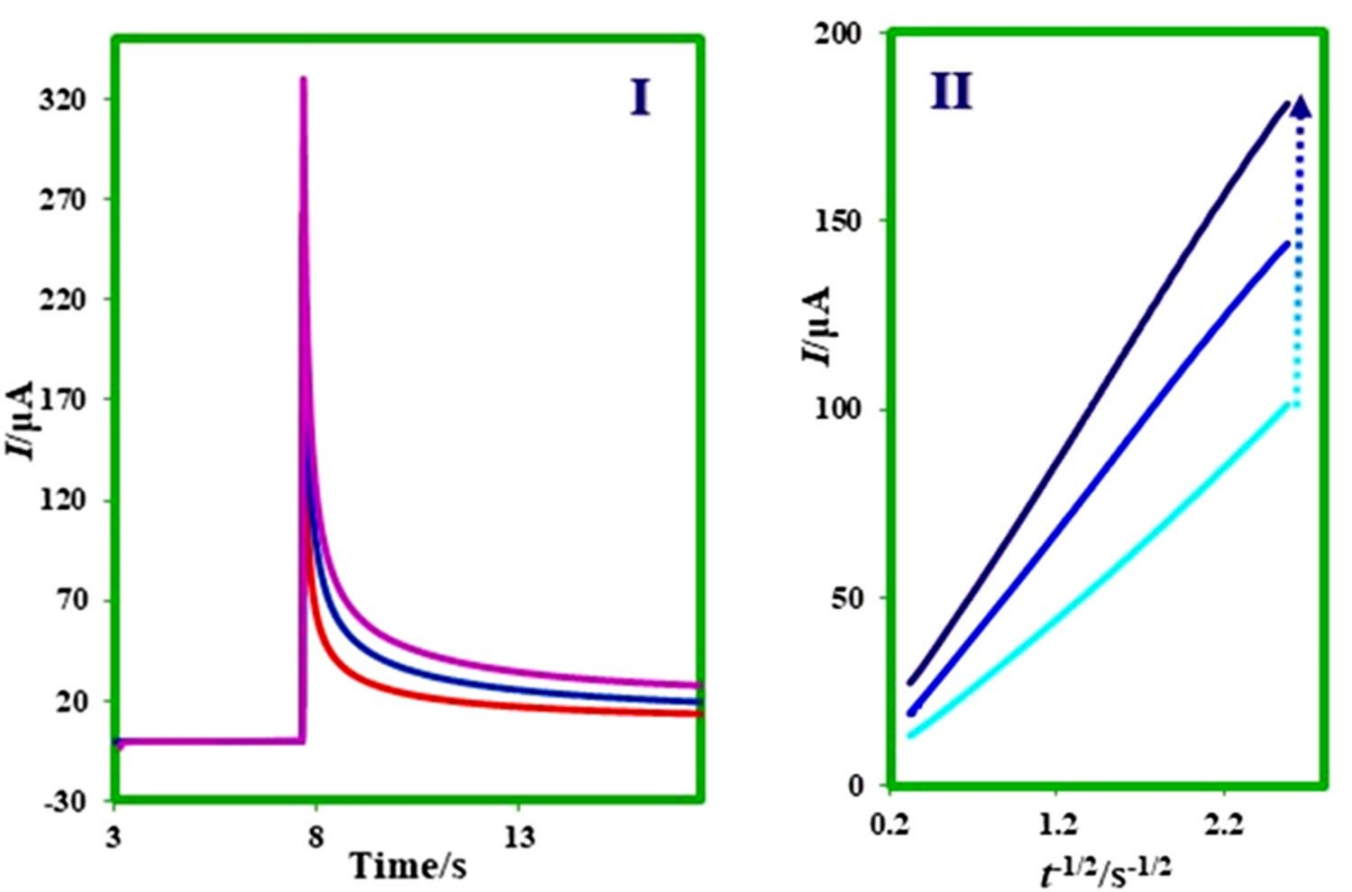
(**I**) Chronoamperograms of **MFA** at the surface of glassy carbon electrode in ethanol containing MgClO_4_ (1.0 M) in various concentrations of **MFA**. Concentrations from a to c are 3.0, 5.0, and 7.0 mM. The potential of 0.95 V versus Ag/AgCl was applied for 10 s. (**II**): *I* *−* *t*^1/2^ plot of **MFA** for the corresponding point in chronoamperogram **I**. Room temperature.

Subsequently, chronoamperograms of **MFA** were recorded in the different concentrations. These chronoamperograms in various concentrations (3.0, 5.0, and 7.0 mM) have been shown in Fig. 8I. Then *I* − *t*^1/2^ diagrams dragged with selecting a curve with the most negligible adsorption effect in Fig. 8II. Finally, after analyzing these diagrams and using the equations proposed by Shoup and Szabo's, the diffusion coefficient of **MFA** has been accounted (1.39 × 10^–5^ ± 0.65 × 10^–6^ cm^2^ s^−1^)^30^.

### Chronocoulometry technique and determination of surface excess, Ӷ (mol cm^−2^), of adsorbed MFA

Chronocoulometry as a helpful technique is applicable for the investigation of the electroactive substance that is adsorbed on the electrode surface. In this technique, the measured total charge (*Q*_total_) in response to the potential step comes from three sources: electrolysis of diffused species (*Q*_d_), electrolysis of adsorbed species (*Q*_ads_), and charging of the double layer (*Q*_dl_)^31^.

$$Q_{total} = 2\pi^{ - 1/2} nFAC^{ * } D^{1/2} t^{1/2} + Q_{dl} + nFA\Gamma^{ * } .$$

In this equation, Ӷ^*^ as surface excess is the amount of adsorbed **MFA** on the surface electrode (mol cm^−2^), and *Q*_dl_ is the capacitive charge. According to the cottrell equation, *Q*_d_ as the diffusion charge is a time-dependence term, but the adsorbed (*Q*_ads_) and double layer (*Q*_dl_) charges are time-independent terms.

**Figure 9:**
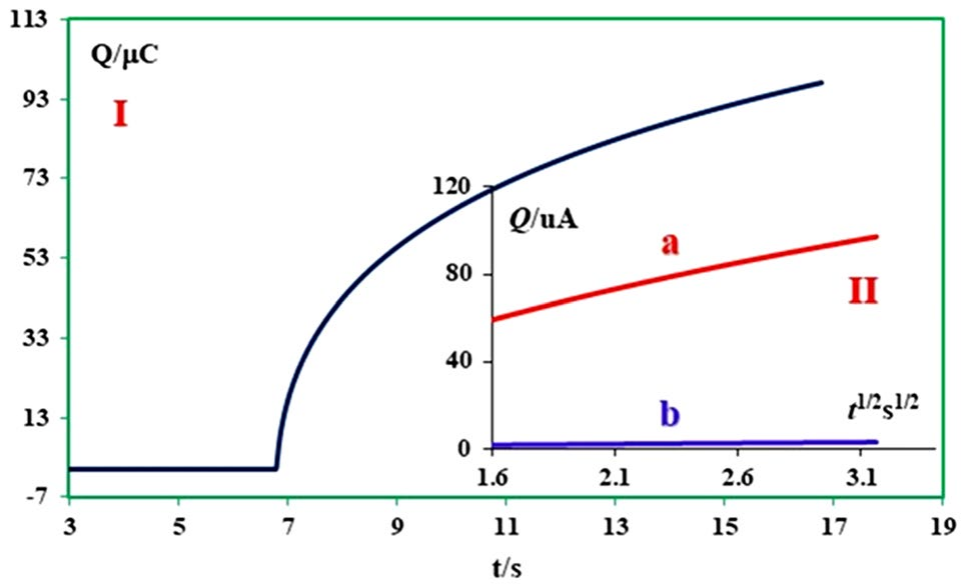
Chronocoulogram of 0.4 mM **MFA** in aqueous buffered solution with pH 9.0. Insets: Curve a: the plot of the total charge (*Q*_total_) vs. *t*^1/2^ of **MFA** (0.4 mM), Curve b: the curve of the total charge (*Q*_total_) vs. *t*^1/2^ of a solution with supporting electrolyte in the absence of **MFA** at the same conditions.

Chronocoulogram of 0.4 mM **MFA** in aqueous solution at pH 9.0 recorded at the surface of the glassy carbon electrode. The plot of total charge (*Q*_total_) vs. t^1/2^ (Anson plot^32^) has been shown in Fig. 9 curve I. Because of the participation of double-layer charging (*Q*_*dl*_) and the electroreduction of adsorbed **MFA** (*Q*_ads_) in the *Q*_total_, the line in Fig. 9 curve II does not pass through the origin. So, the interrupt of this line can be used to designate the sum of *Q*_*ads*_ and *Q*_*dl*_^33^. Moreover, *Q*_*dl*_ obtained of chronocoulogram of blank solution. The intercept in the plot of Anson *Q*–*t*^1/2^ for blank solution illustrated of *Q*_*dl*_*.* *Q*_*ads*_ is calculated by subtracting *Q*_dl_ from the intercept in Fig. 9, II. Finally, with having A and Ӷ* as the amount of adsorbed **MFA**, 0.01 µmol cm^−2^ was calculated.

### Electrochemical oxidation of MFA in the presence of sodium nitrite (NaNO_2)_

**Figure 10:**
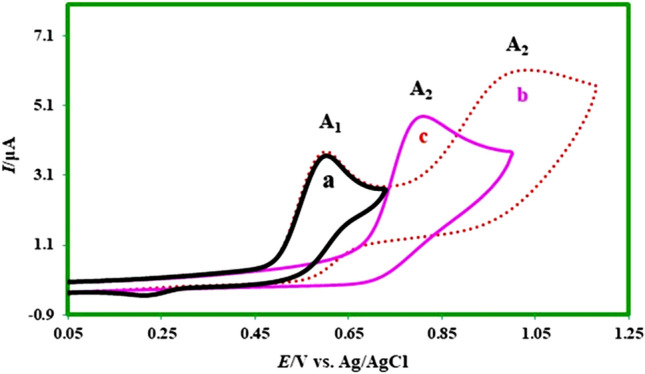
Cyclic voltammograms for a solution of (a) 0.4 mM **MFA** (b) 0.4 mM **MFA** in the presence of 0.4 mM sodium nitrite (c) 0.4 mM sodium nitrite at a glassy carbon electrode, in phosphate buffer solution (c = 0.2 M, pH 7.0)/ethanol (70/30 v/v). Scan rate: 50 mV s^−1^. Room temperature.

The cyclic voltammogram of the mefenamic acid (**MFA**) (0.4 mM) in buffered solution with pH = 7.0 (c = 0.2 M) is illustrated in Fig. 10. This voltammogram (Fig. 10a) contains an anodic peak A_1_ at the potential of 0.8 V vs. Ag/AgCl, which is related to the oxidation of **MFA** to its oxidized form. Figure 10, curves b and c show the cyclic voltammograms of **MFA** in the presence of 0.4 mM of sodium nitrite and 0.4 mM sodium nitrite in the absence of **MFA** consequently.

**Figure 11:**
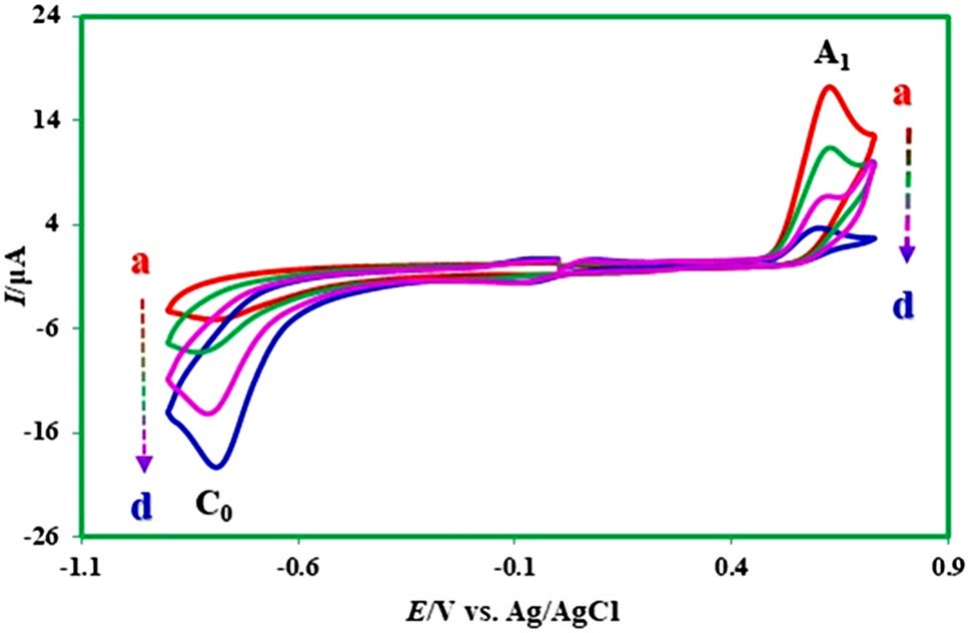
Cyclic voltammograms of 0.25 mmol **MFA** in the presence of 2.5 mmol ion nitrite, during controlled potential coulometry at 0.5 V versus Ag/AgCl. After consumption of: (a) 0, (b) 35, (c) 70 and (d) 100 C in Scan rate 50 mV s^−1^; Room temperature.

As can be seen (Fig. 10 curves a and c), anodic oxidation of **MFA** and sodium nitrite takes place in the different potentials and hence this allows for investigating of the possibility of the chemical reaction between the product of electrooxidation of **MFA** with sodium nitrite. In the following, controlled-potential coulometry was performed to obtain information about the electrooxidation of **MFA** and the chemical reaction of its product with ion nitrite. Figure 11 shows cyclic voltammograms of 0.25 mmol of **MFA** and 2.5 mmol of nitrite ion recorded during the controlled-potential coulometry at 0.5 V versus Ag/AgCl (water (0.2 M phosphate buffer, pH 7.0)/ethanol (70:30 v/v).

Cyclic voltammograms analysis (Fig. 11) shows the progressive formation of a new cathodic peak (C_0_), parallel to the disappearance of the anodic peak A_1_. These voltammetric observations and other information from spectroscopic methods such as ^1^H NMR, ^13^C NMR, FT-IR, and molecular mass allow us to propose the production of nitromefenamic acid as the final product in the electrolysis cell.

**Figure 12:**
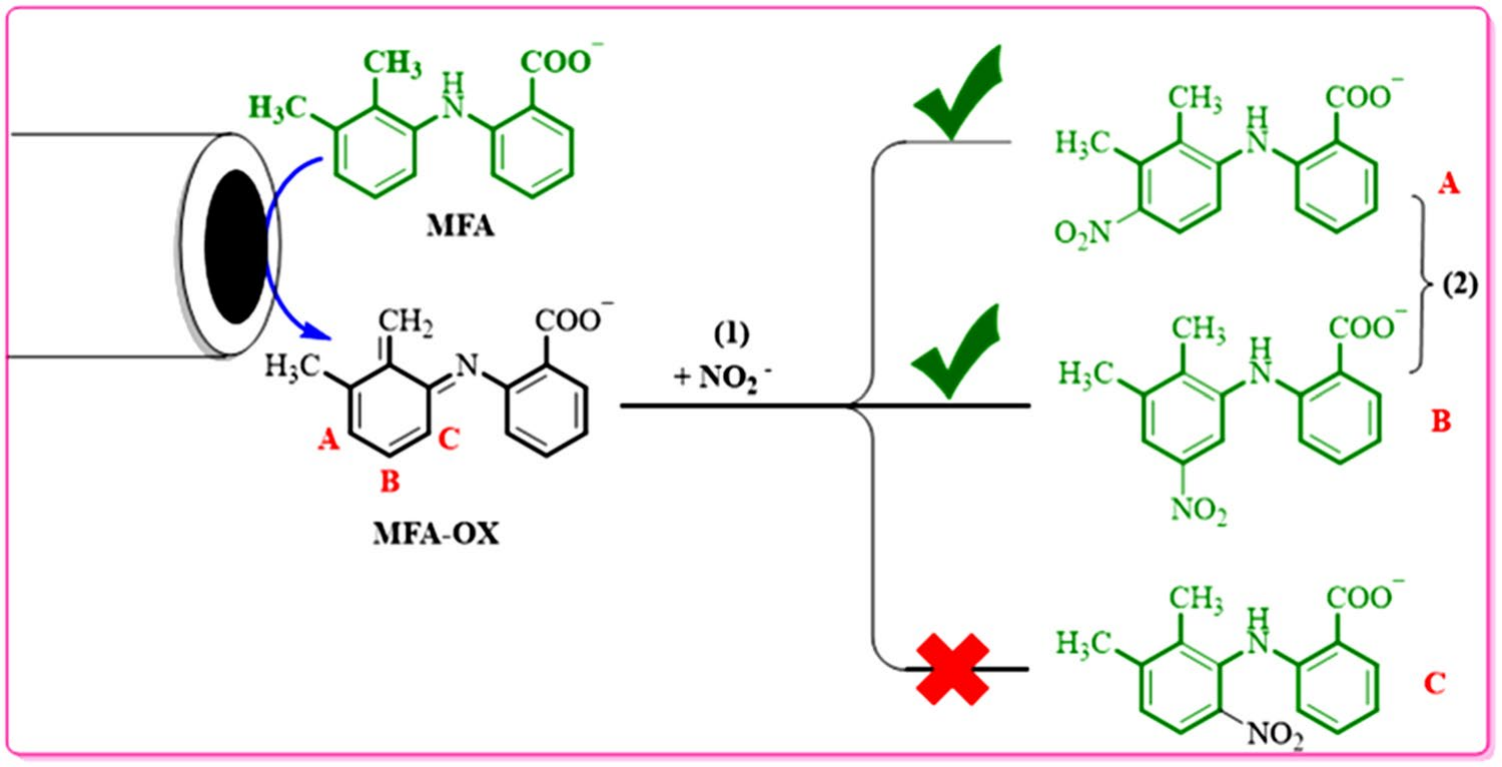
Different pathways of nucleophilic addition reaction of MFA-OX.

The generation of nitromefenamic acid followed by a Michael-type addition of nitrite ion (**1**) with the oxidation form of **MFA**. Experimental ^1^H NMR for the final product is indicative of the synthesized two isomers with the 57% and 32% isolated yield in the electrolysis cell (A and B in Fig. 12).

### Computational studies

**Figure 13:**
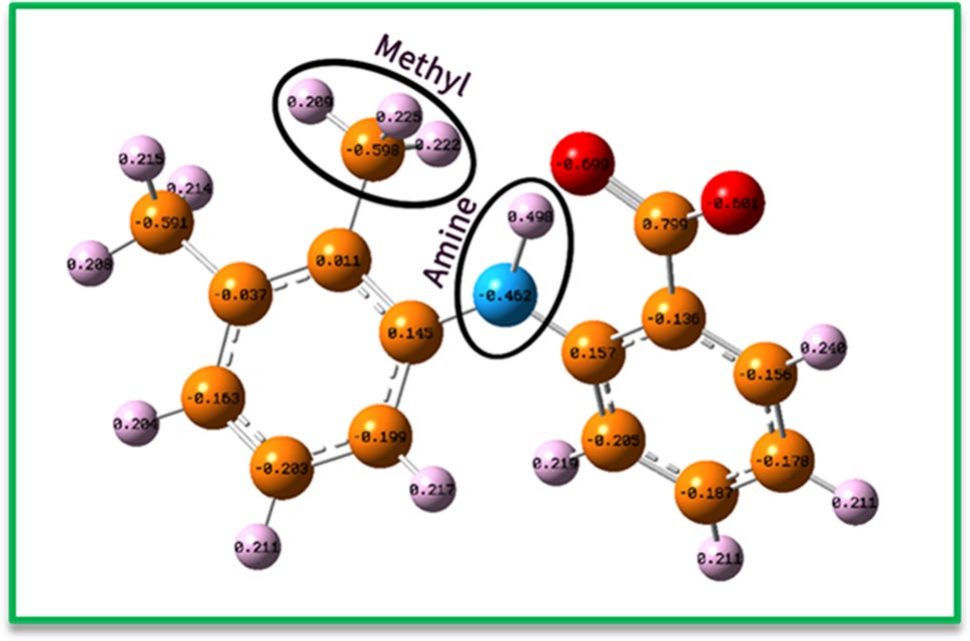
The natural charge distribution of **MFA** at B3LYP/6–311 + G (2d, p) level of theory.

In this section of research, computational studies are performed to get better information about the structure of final products and to confirm the electrochemical mechanism in the previous part. Therefore, different parameters were investigated respectively: (1) The distribution of partial charge, which was used to approve the oxidation mechanism of **MFA** and also to determine the best active site as a nucleophile acceptor in the oxidized form of **MFA**. (2) Thermodynamic stability of intermediates, which was verified using the relative Gibbs free energy^34^. (3) The Kinetic stability of intermediates was evaluated via the HOMO–LUMO energy gap^24^. Optimization of structures and all the mentioned calculations were fully performed using Density Functional Theory (DFT) and the B3LYP/6–311 + G (2d, p) basis set^35^. The distribution of partial charge in the optimized geometry of **MFA** shows that this molecule can become an electrophile by losing protons from the most negative atoms. The distribution of partial charge of nitrogen and carbon atoms (− 0.462e and − 0.598e) in the amine and methyl group respectively, can perfectly approve that they are the best electronegative atoms to losing the protons, among others (Fig. 13).

In the next step, the natural charge was considered in the none-aromatic ring of **MFA-OX** to detect the most positive atoms for the nucleophilic addition^24^. As shown in Fig. 12, the partial charge of sites A, B, and C are − 0.231e, − 0.128e, and − 0.246e, respectively.

Based on these results, the NO_2_—will attack the A and B sites. The reaction will never occur in path C (Fig. 12) because: (a) In the **MFA-OX**, atom C is not in the Meta position of oxidized functional groups. Hence it is not an active site to accept the electrons as much as other positions. (b) The addition of NO_2_^-^ to this site cannot restore the aromaticity.

**Figure 14:**
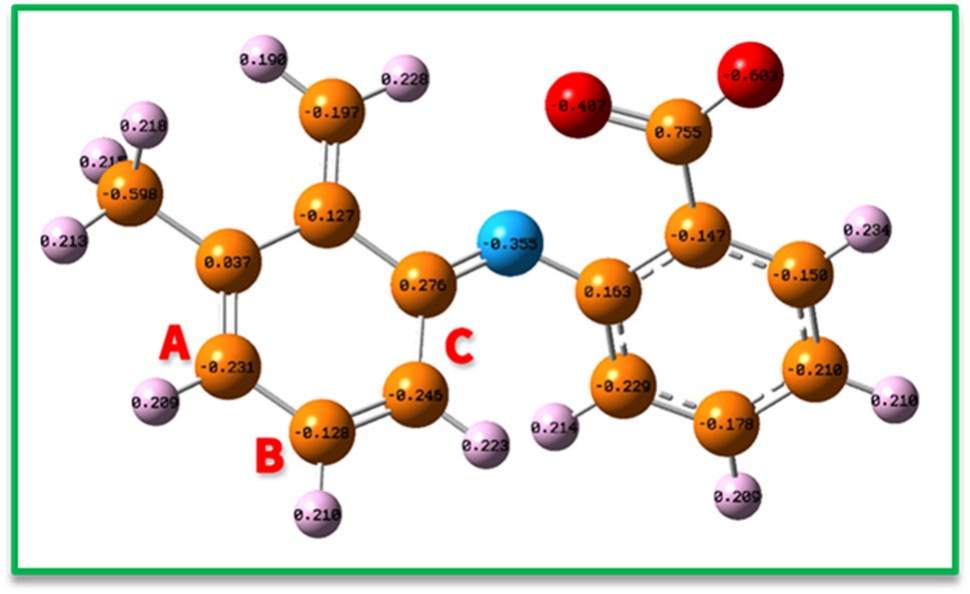
The natural charge distribution of **MFA-OX** at B3LYP/6–311 + G (2d, p) level of theory.

**Figure 15:**
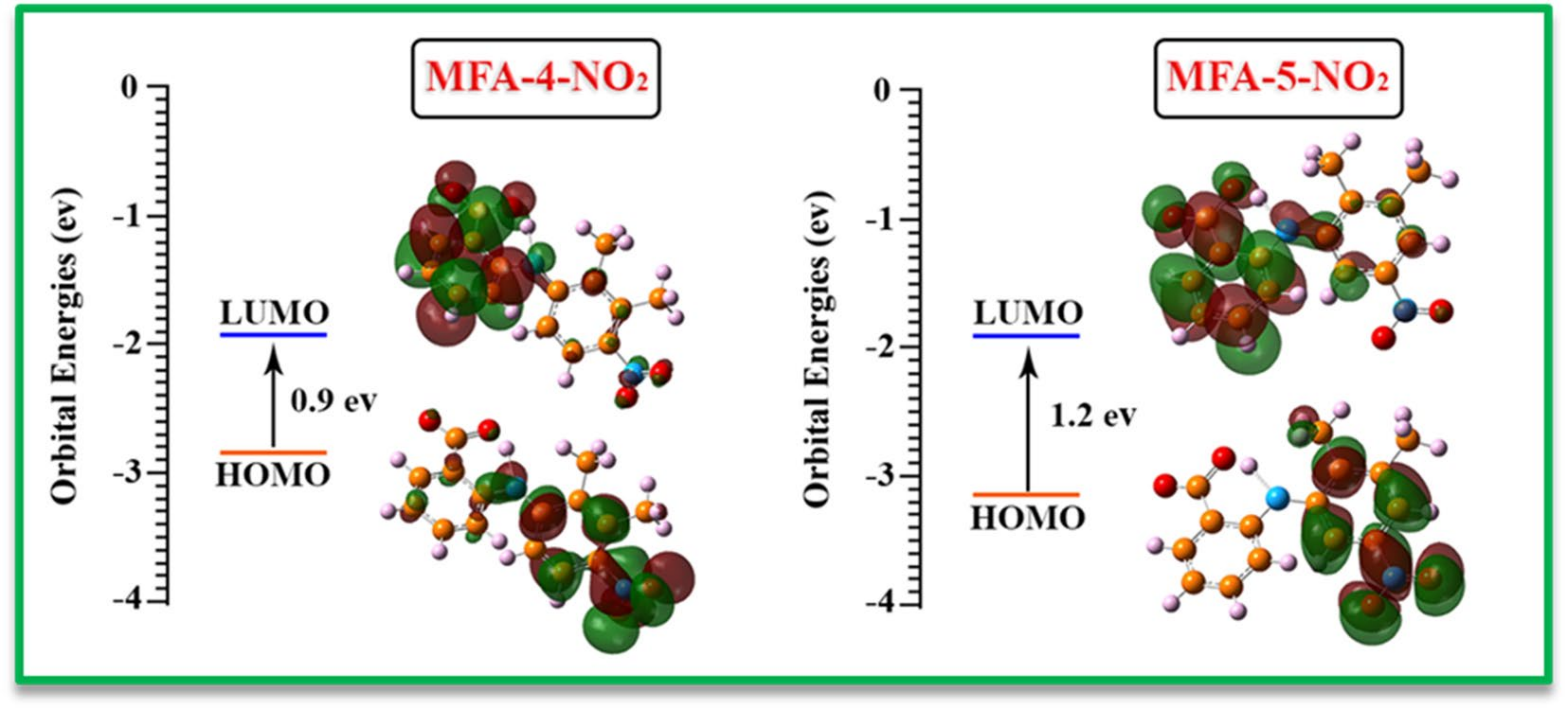
HOMO–LUMO energy gaps of possible intermediates.

In the following, the thermodynamic stability of possible intermediates (Int) was compared using Gibbs free energy (Fig. 14). The relative Gibbs free energies for **A** (MFA-4-NO_2_**)** and **B** (MFA-5-NO_2_) are 0.55 and 0.0 kcal/mol, and In addition, the calculated HOMO–LUMO energy gaps for the mentioned molecules are 0.9 and 1.2 eV, respectively (Fig. 15). The result perfectly denotes that Int B is slightly more stable than Int A in both thermodynamic and the kinetic perspective; however, this difference is diminutive and can be ignored in the experimental conditions.

Eventually, by insights drawn from this theoretical analysis and other spectroscopic data (^1^HNMR spectrum), we can conclude that A and B molecules (Fig. 14) are the final products isolated from the electrolysis cell.

### Electrochemical behavior of nitro mefenamic acid (2)

**Figure 16:**
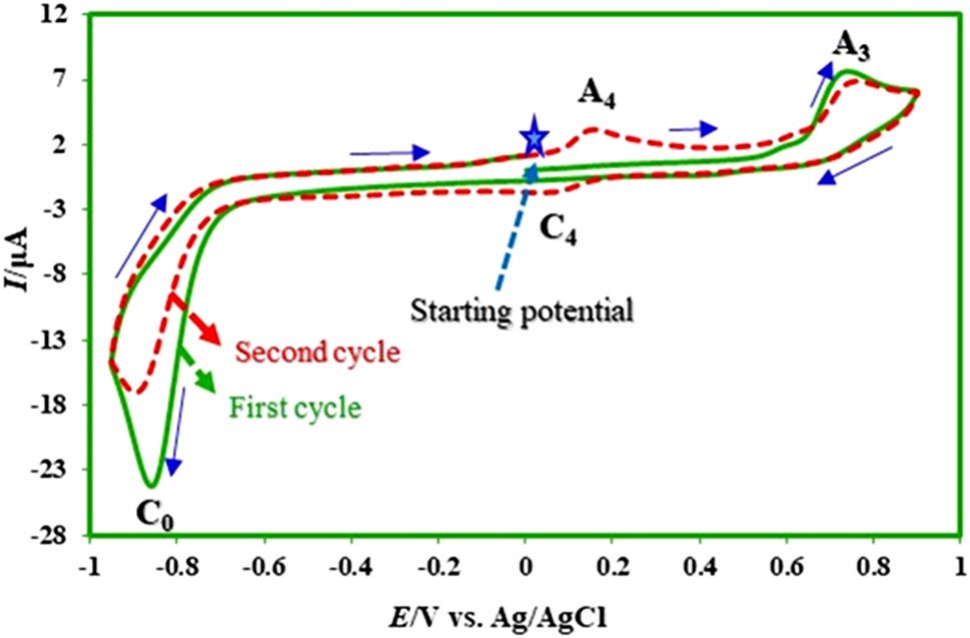
Cyclic voltammograms (first and second cycle) for a saturated solution of nitromefenamic acid at a glassy carbon electrode, in phosphate buffer solution (c = 0.2 M, pH 7.0); scan rate: 100 mV s^−1^; room temperature.

**Figure 17:**
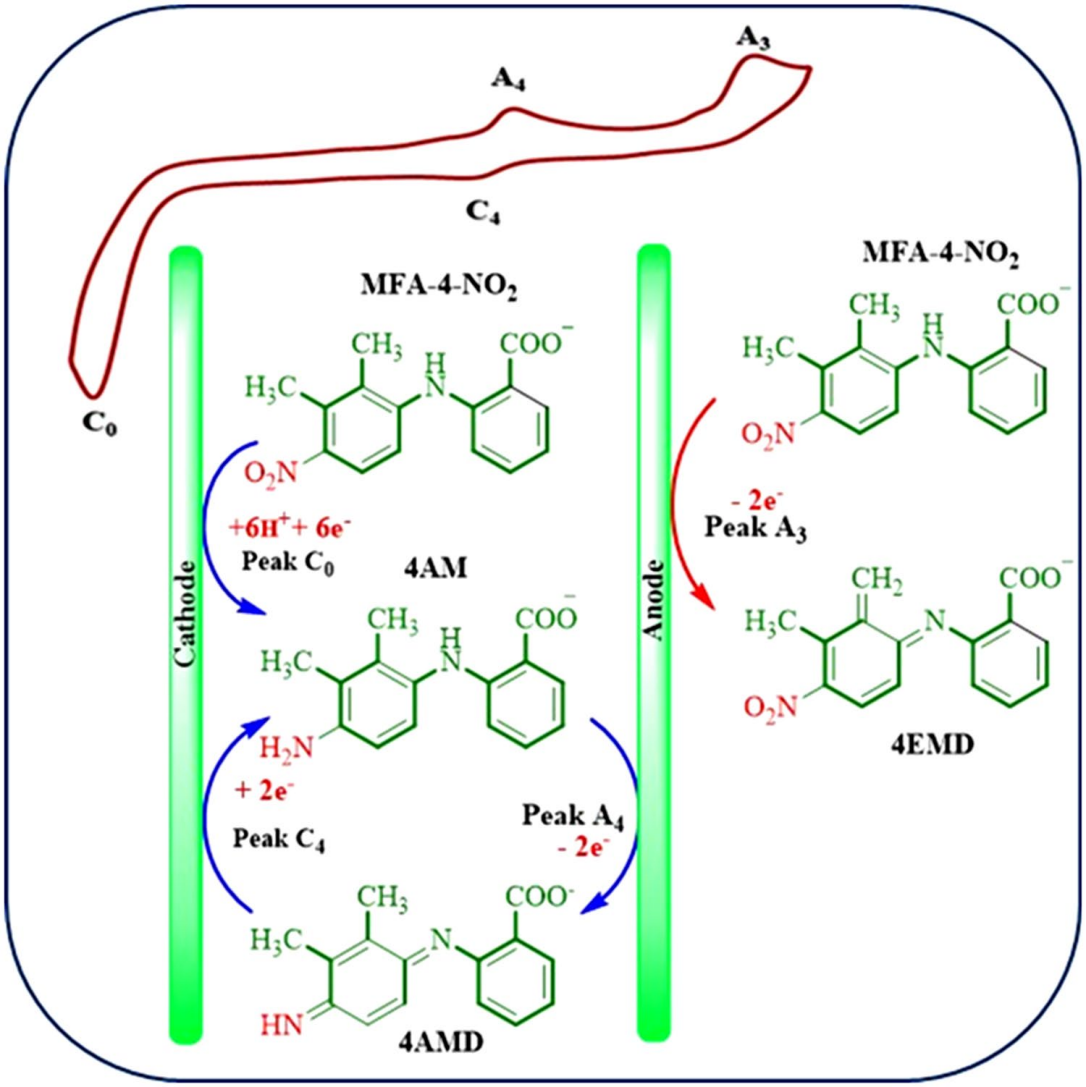
The proposed mechanism for oxidation–reduction of nitromefenamic acid.

The electrochemical behavior of produced nitromefenamic acids (**2**) has been investigated by cyclic voltammetry as a practical technique. Figure 16 shows the cyclic voltammograms of **2** in the positive and negative-going scans. In the positive-going scan, the voltammogram shows: (a) one anodic peak (A_3_) at 0.70 V vs. Ag/AgCl, which correspond to the transformation of **2** to oxide form (**4EMD**, **5EMD**) (Fig. 17), with an irreversible feature, (b) an irreversible cathodic peak (C_0_) corresponding to the reduction of **2** to **4AMD**, **5AMD** and (c) a redox couple (A_4_/C_4_) that appears in the second cycle at less positive potentials than that the A_3_ peak. The oxidation of **2** (A_3_) is happening at more positive potentials than that the oxidation of **MFA** by virtue of the nitro group. To investigate the relationship between the A_3_ peak and A_4_/C_4_ couple, the potential was scanned in the negative-going scan from 0.35 V to − 0.9 V vs. Ag/AgCl. The voltammogram shows one cathodic peak (C_0_) and a redox couple (A_4_/C_4_) at less positive potentials than the A_3_ peak. The pathway is leading to the oxidation/reduction peaks A_4_/C_4_ is given in Fig. 17.

Anodic peak (A_4_) at 0.20 V versus Ag/AgCl corresponds to the oxidation of cathodically generated **4-AM** and **5-AM** (in C_0_ peak) to **4-AMD** and **5-AMD** (Fig. 17). As can be seen, the A_4_ and C_4_ peaks appear without oxidation of **2**.

**Figure 18:**
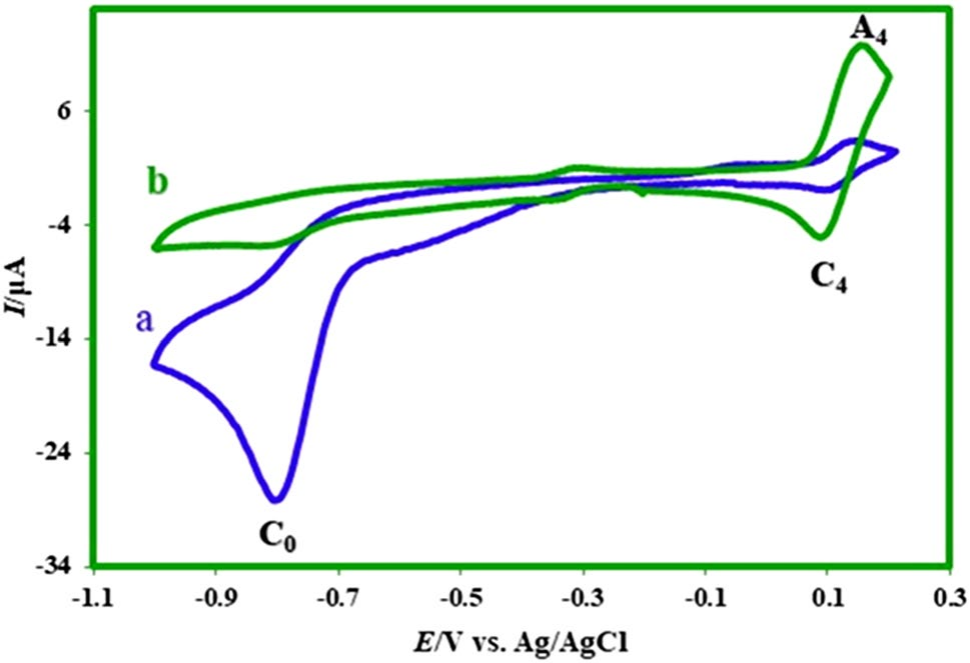
Cyclic voltammograms of 0.2 mmol nitro mefenamic acid in the controlled potential coulometry at − 0.8 V versus Ag/AgCl after consumption of: (a) 0 and (b) 120 C in phosphate buffer solution (c = 0.2 M, pH 7.0); Scan rate 100 mV s^−1^; Room temperature.

**Figure 19:**
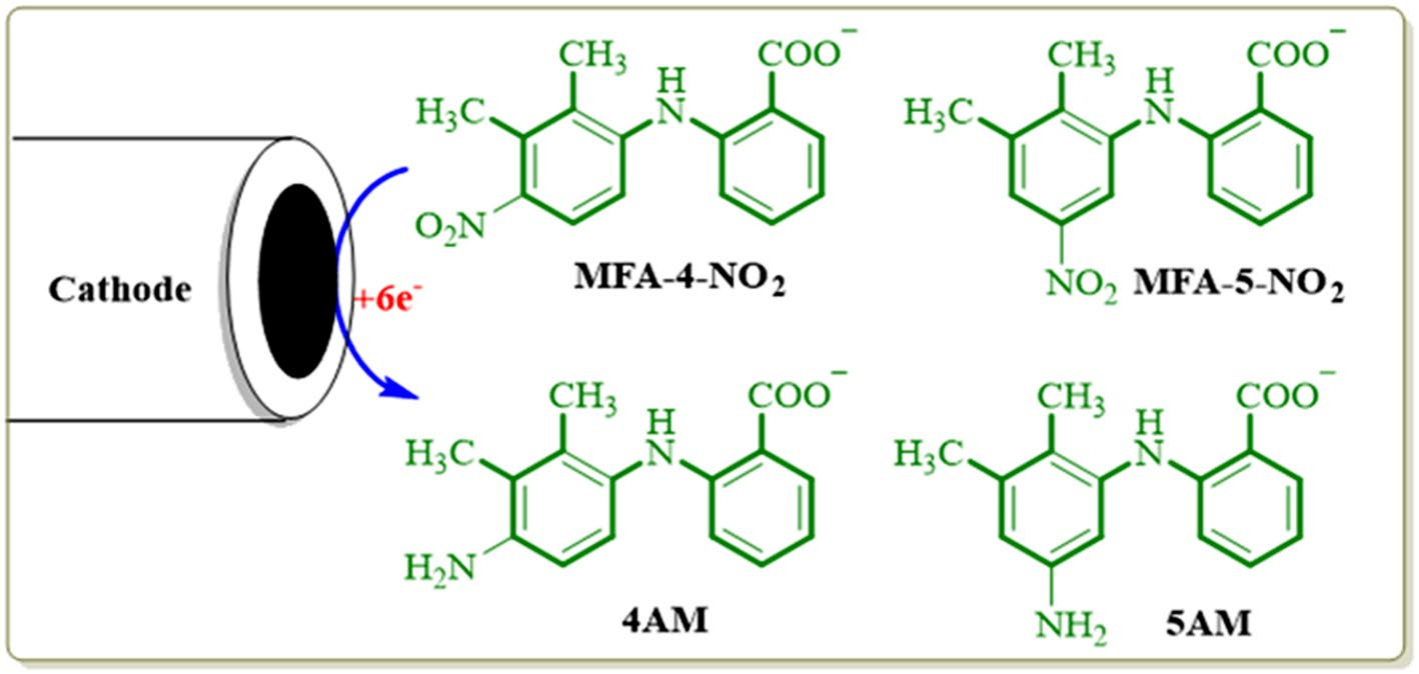
Reduction of nitromefenamic acid at the surface of glassy carbon electrode.

Controlled-potential coulometry was used to prove the pathway in Fig. 17 and also to synthesis new products with the amine group. Figure 18 shows cyclic voltammograms recorded in the controlled-potential coulometry at first and after consumption of 120 C in aqueous solution (0.2 M phosphate buffer, pH 7.0) containing 0.2 mmol of produced nitro mefenamic acid (**2**) at − 0.8 V versus Ag/AgCl. Cyclic voltammograms (Fig. 18, curves a, b) shows the formation of a new redox couple (C_4_/A_4_) simultaneous to the disappearance of the cathodic peak C_0_. The cathodic peak (C_0_) disappears when the charge consumption becomes about 6e^-^ per molecule of **2** (two isomers in Fig. 14). These observations allow us to confirm the pathway in Fig. 17. At the end of electrolysis, the solution's pH reached 3.0 by adding hydrochloric acid solution, and then the product was extracted using ethyl acetate. After extraction, the substance was concentrated and then dried. Obtained orange residue characterized by M.p. and IR. The elimination of –NO_2_ and observation of –NH_2_ group in the IR spectrum is indicative of the formation of product **3** (Fig. 19).

### Diazotization and diazo coupling reaction

It has been already confirmed that diazotization of amines leads to the production of diazonium salts^36,37^. In this stage to the preparation of diazonium salts, 0.25 mM of the synthesized amines (**3**) diffused in water and hydrochloric acid and placed in an ice bath at a temperature of less than 5 °C. Sodium nitrite was added to this solution in equal proportions and eventually led to the synthesis of diazonium salts. In the following, 0.25 mmol of β-naphthol was added to the diazonium salt as a coupler species. The coupling reaction was performed at a temperature below 10 °C. The final solution was filtered, and the residual precipitate was rinsed with distilled water. The two azo products were well separated and purified by column chromatography (silica gel) with several solvent systems. At the first stage, the solvent system was ethyl acetate/n-hexane/chloroform with a volume ratio 25/25/50 respectively and in the second stage, the column run with chloroform.

**Figure 20:**
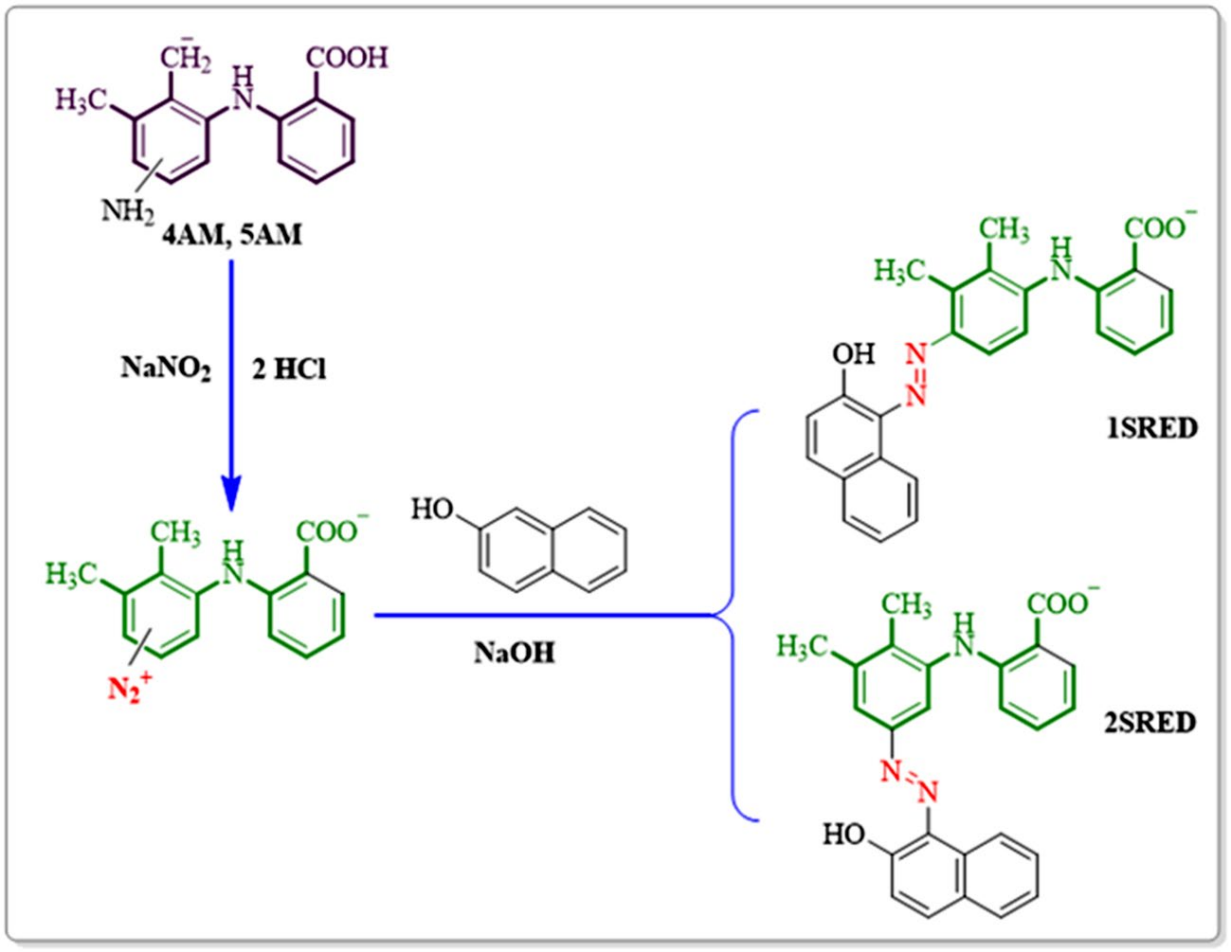
Diazotization and azo coupling.

After separation and purification, the products was identified using spectroscopic methods: FT-IR, ^1^H NMR, ^13^C NMR, and MS. Based on the observed results, Fig. 20 presented for the synthesis of two diazo compounds.

### Evaluation of dyeing properties of synthetic products

#### Dyeing properties of nitromefenamic acid (2)

**Figure 21:**
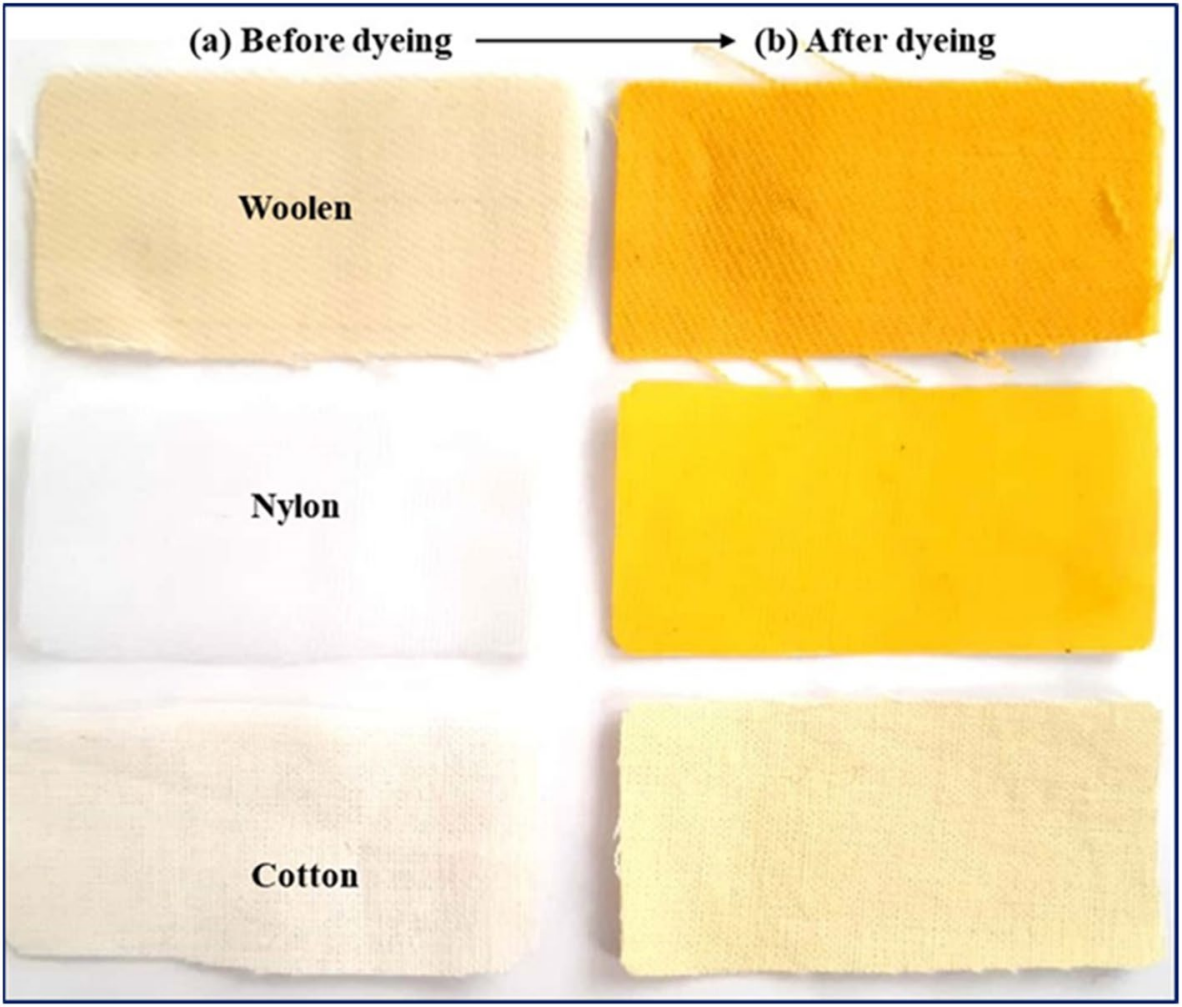
Results of the dyeing process of produced nitromefenamic acid (**2**) for different fabrics: (**a**) before dyeing and (**b**) after dyeing.

The observation of the high color intensity of synthesized products prompted us to investigate the dyeing properties of these products. At first, dyeing testing performed on the different fabrics. First, 100 ml of produced nitromefenamic acid 1% w/v solution prepared, then the colored bath provided at a concentration of 1% and at a liquor ratio, LR, 30:1. Liquor ratio (LR) is the ratio of the bath volume to the weight of the textile material (2 g) (ml/g or l/kg)^38^. An acetic acid solution of 3% was added to the color bath. The fabrics were immersed in the bath at 40 °C. Then the temperature increased at a steady rate, and the fabrics rotated at 98 °C for 45 min. After finishing the dyeing process, the fabrics were washed with cold water and dried at room temperature. The results in the Fig. 21 show that dyeing on nylon and wool fabrics has the highest yield and synthetic dye is among the acidic dyes of textiles.

### Dyeing properties of 1S-RED and 2S-RED diazo products

**Figure 22:**
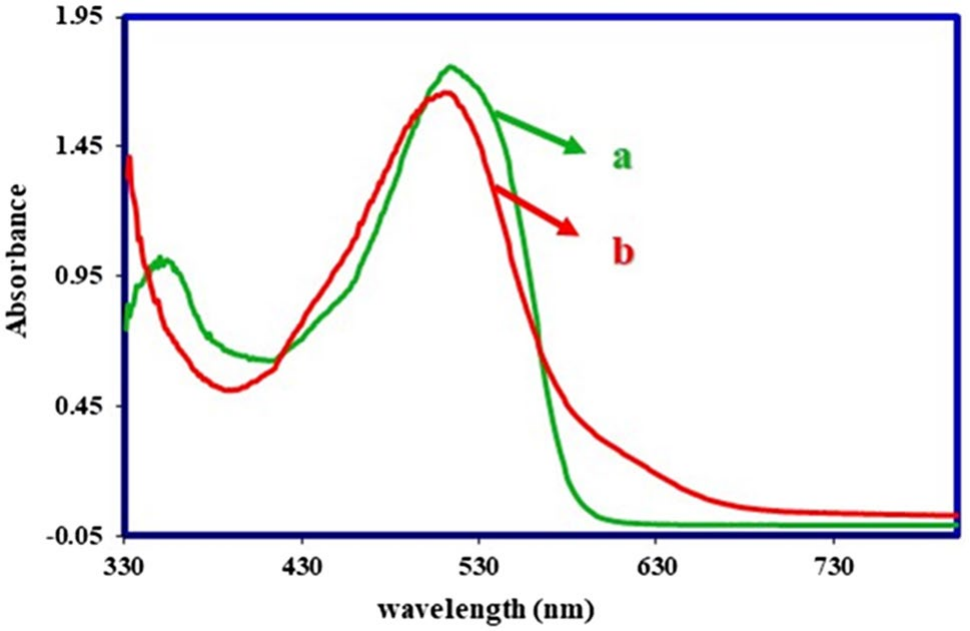
Ultraviolet / Visible absorption spectrum (a) Solvent Red 4 (b) a solution of mixture **1S-RED** and **2S-RED** in acetone; Room temperature.

**Figure 23:**
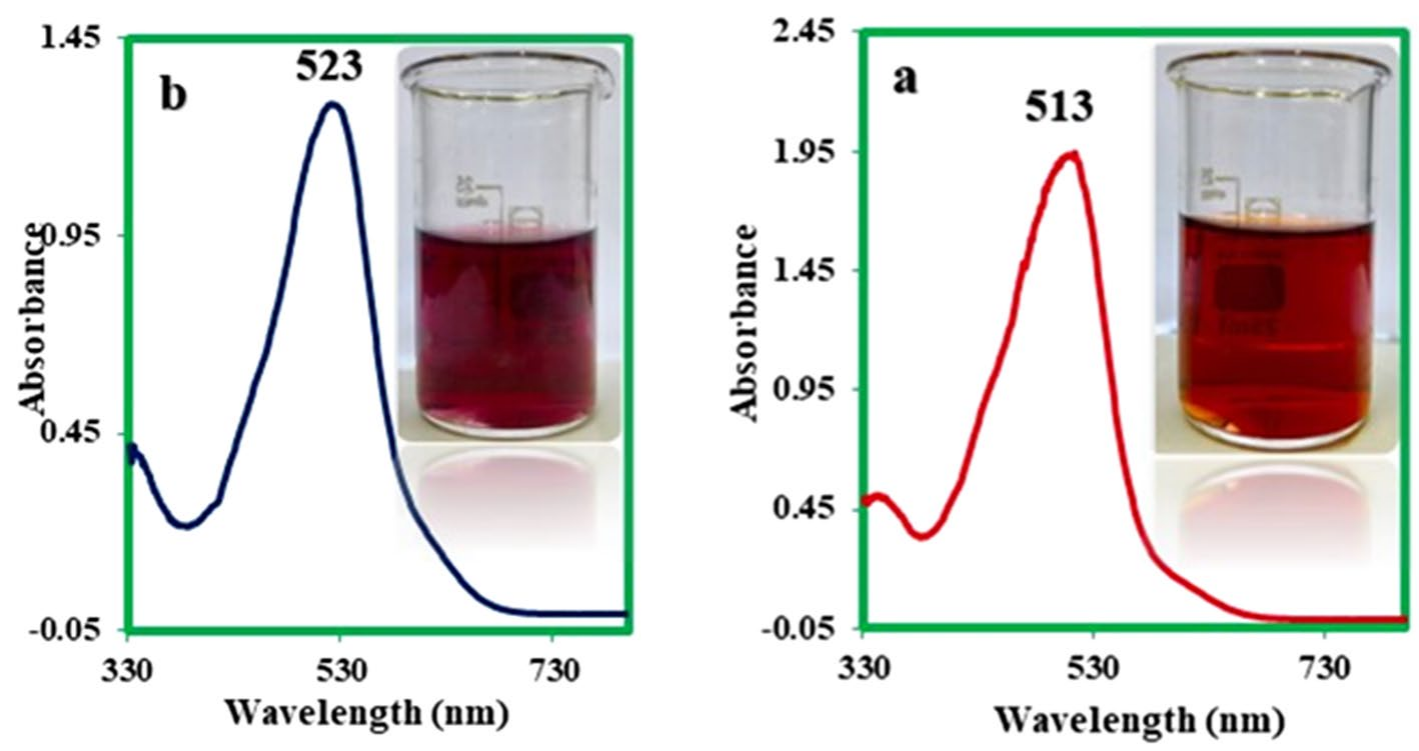
Ultraviolet / Visible absorption spectra (**a**) **1S-RED** and (**b**) **2S-RED** solution separately in acetone. Room temperature.

Since the two diazo products **1S-RED** and **2S-RED** are a part of the solvent-based dyes and insoluble in water, therefore, dyeing testing did not perform on the fabric. The solubility, strength, and color shades were examined for these products. It was approved that the results of the color were similar to the Solvent Red 4 (a commercially recognized color). As shown in Fig. 22, the synthetic dye has an absorption peak with λ_max_ = 514 nm similar to the reference color absorption spectrum. The absorption spectrum of **1S-RED** and **2S-RED** is taken individually. The results in Fig. 22 showed that **1S-RED** has an absorption peak at λ_max_ = 523 nm, and **2S-RED** has an absorption peak at λ_max_ = 513 nm. The **2S-RED** solution has a peak absorption at a wavelength higher than the **1S-RED**, which corresponds to its red–purple color. Besides, the comparison of the absorption peaks of **1S-RED** and **2S-RED** with the absorption peak reported for **2**, it can be concluded that the increasing length of the maturation system has changed the red location of the absorption peaks (Fig. 23).

## Conclusions

In this electrochemical study, the electrooxidation of mefenamic acid (**MFA**) was carried out with details in various pH values by differential pulse voltammetry. Based on our results, the oxidation of **MFA** is highly dependent on pH, under the *E*_ir_ mechanism. The diffusion coefficient and the surface excess, Ӷ* of **MFA** in aqueous buffered solution, determined by using the single potential-step chronoamperometry and chronocoulometry methods. the Electrochemical nitration of **MFA** in an aqueous solution and the presence of nitrite ion (**1**) carried out and two new nitromefenamic acid derivatives has been synthesized. Our results indicate that the oxidized form of **MFA** participates in a Michael-type addition reaction with nitrite ion (**1**) to form the corresponding Nitromefenamic acids (**MFA-4-NO**_**2**_ and **MFA-5-NO**_**2**_). The electrochemical reduction of produced nitromefenamic acids was investigated and eventually, two new azo derivatives have been generated via electroreduction of produced nitromefenamic acids and conduction of diazotization reaction, respectively. These new nitro and azo derivatives approved as paints and can be applicable in textile and dyeing industry. Also, the theoretical studies were in accordance with the experimental observations and approved the suggested mechanism for the electrochemical oxidation of mefenamic acid in the presence of nitrite ion (**1**) as a nucleophile.

## Materials and methods

### Apparatus and reagents

Reaction equipment said in an earlier paper^10,39^. All chemicals were reagent-grade materials from E. Merck. These chemicals are used without further purification.

### Electrochemical synthesis of nitromefenamic acid

A solution of phosphate buffer (ca. 100 ml; c = 0.2 M, pH = 7.0) in water/ethanol (70:30 v/v), containing mefenamic acid (**MFA**) (0.25 mmol) and sodium nitrite (2.5 mmol) (**1**) was electrolyzed in a divided cell at 0.5 V vs. Ag/AgCl. The electrolysis was terminated when the current decreased by more than 95%. At the end of electrolysis, after acidification of the solution with hydrochloric acid, the precipitated solid was washed several times with cold water. After washing and drying, products characterized by M.p., IR, ^1^H NMR, ^13^C NMR, and MS.

### Computational methods

The computational studies were performed using the Gaussian09 package of programs^40^. All the optimizations of geometries were fully carried out using DFT method at B3LYP level and 6–311 + G (2d, p) basis set for desired molecules. The Natural Bond Orbital (NBO) analysis, vibration frequencies, and the orbital energies of the investigated compounds were also calculated at the same level of theory^24^.

## Characterization of products

### MFA-4-NO_2_: 2-((2,3-dimethyl-4-nitrophenyl)amino)benzoic acid (C_15_H_14_N_2_O_4_)


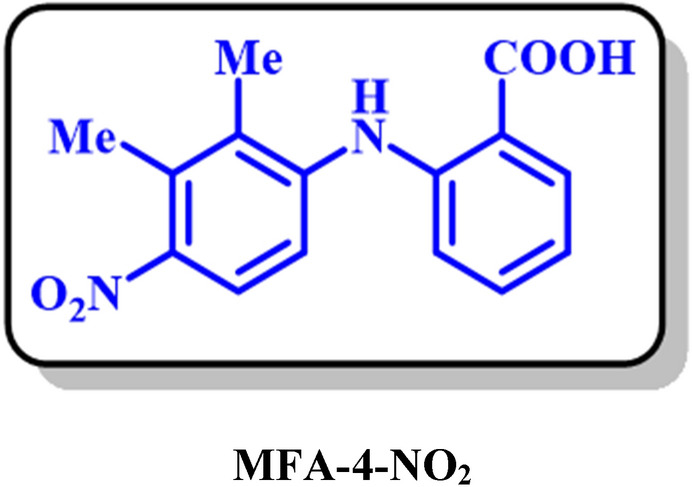


Isolated yield: 57%. M.p.: 278–281. ^1^H NMR (500 MHz, DMSO-d6) δ (ppm): 2.27 (s, 3H, aliphatic), 2.44 (s, 3H, aliphatic), 6.81 (t, *J* = 8.0 Hz, 1H, aromatic), 7.13 (d, *J* = 7.1 Hz, 1H, aromatic), 7.24 (t, *J* = 7.5 Hz, 1H, aromatic), 7.35 (d, *J* = 8.8 Hz, 1H, aromatic), 7.74 (d, *J* = 8.8 Hz, 1H, aromatic), 7.94 (d, *J* = 7.8 Hz, 1H, aromatic). ^13^C NMR (125 MHz, DMSO-d6) δ (ppm): 14.1 (C-15), 16.5 (C-13), 110.9 (C-6), 115.7 (C-9), 119.2 (C-4), 123.3 (C-10), 129.3 (C-3), 131.7 (C-5), 126.1 (C-12), 146.5 (C-7), 142.9 (C-11), 111.2 (C-2), 116.4 (C-14), 125.9 (C-8), 169.6 (C-1). IR (KBr) *ν* (cm^−1^): 3243 (N–H, O–H), 3075, (weak, C–H, aromatic), 2924, 2855 (weak, C–H, aliphatic), 1670 (weak C=O), 1497 (strong, C=C), 1578, 1383 (strong, N=O), 1297 (strong, C–O), 1171, 751. MS (EI, 70 eV): *m/z* (relative intensity) 286 (M^+^·, 45), 222 (40), 167 (64), 149 (100), 76 (50).

### MFA-5-NO_2_: 2-((2,3-dimethyl-5-nitrophenyl)amino)benzoic acid (C_15_H_14_N_2_O_4_)


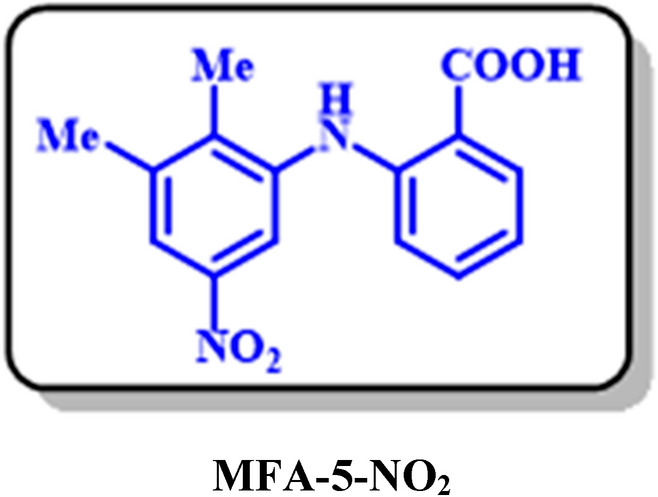


Isolated yield: 32%. M.p.: 278–281. ^1^H NMR (500 MHz, DMSO-d6): δ 2.28 (s, 3H, aliphatic), 2.34 (s, 3H, aliphatic), 6.65 (t, *J* = 8.65 Hz, 1H, aromatic), 7.68 (d, *J* = 8.0 Hz, H, aromatic), 6.58 (t, *J* = 8.0 Hz, H, aromatic), 76.98 (t, *J* = 7.9 Hz, 1H, aromatic), 7.86 (d, *J* = 7.9 Hz, 1H, aromatic), 7.02 (t, *J* = 7.0 Hz, 1H, aromatic). ^13^C NMR (125 MHz, DMSO-d6): δ 15.08 (C-15), 20.46 (C-13), 110.9 (C-6), 119.24 (C-4), 131.72 (C-5), 126.14 (C-11), 129.29 (C-3), 132.92 (C-8), 129.6 (C-9), 146.49 (C-7), 141.77 (C-12), 111.16 (C-2), 126.35 (C-14), 125.99 (C-8), 169.65 (C-1). IR (KBr) *ν* (cm^−1^): 3243 (broad, N–H, O–H), 3075, (weak, C–H, aromatic), 2924, 2855 (weak, C–H, aliphatic), 1670 (weak C=O), 1497 (strong, C=C), 1578, 1383 (strong, N=O), 1297 (strong, C–O), 1171, 751. MS (EI, 70 eV): *m/z* (relative intensity) 286 (M^+^·, 45), 222 (40), 167 (64), 149 (100), 76 (50).

### 1S-RED: 2-((4-((2-hydroxynaphthalen-1-yl)diazenyl)-2,3-dimethylphenyl) amino)benzoic acid (C_25_H_20_N_3_O_3_)


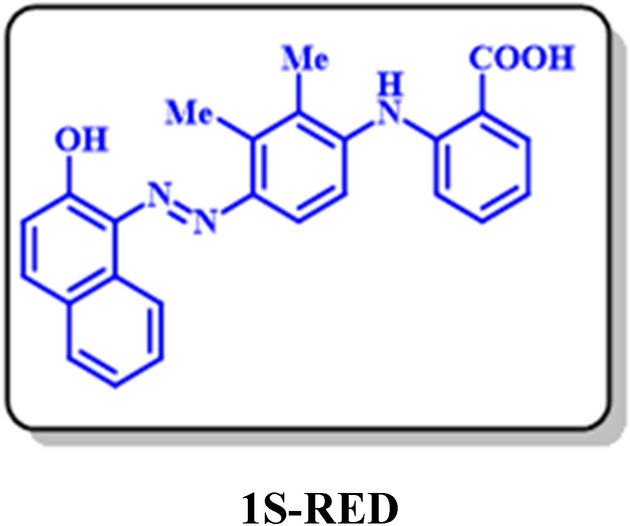


Isolated yield: 46%. Mp > 300 °C (Dec.). ^1^H NMR (500 MHz, CD_3_OD) δ (ppm): 2.35 (s, 3H, aliphatic), 2.53 (s, 3H, aliphatic), 6.79 (t, *J* = 8.0 Hz, 1H, aromatic), 7.01 (d, *J* = 8.9 Hz, 1H, aromatic), 7.19 (d, *J* = 8.9 Hz, H, aromatic), 7.56 (t, *J* = 7.6 Hz, 1H, aromatic), 7.72 (d, *J* = 8.3 Hz, 1H, aromatic), 7.82 (d, *J* = 9.0 Hz, 1H, aromatic), 7.92 (d, *J* = 9.2 Hz, 1H, aromatic), 7.98 (d, *J* = 7.8 Hz, 1H, aromatic), 8.7 (d, *J* = 8.5 Hz, 1H, aromatic), 8.55 (s, 1H, aliphatic). ^13^C NMR (125 MHz, CD_3_OD) δ (ppm): 18.82 (C-15), 29.43 (C-13), 113.98 (C-6), 115.02 (C-9), 116.75 (C-4), 117.73 (C-10), 121.19 (C-24), 121.7 (C-20), 124.58 (C-19), 127.9 (C-21), 128.15 (C-18), 130.5 (C-3), 131.66 (C-5), 136.61 (C-23), 122.5 (C-2), 127.76 (C-14), 128.11 (C-17), 129.63 (C-16), 132.08 (C-22), 133.13 (C-13), 139.84 (C-16), 142.67 (C-11), 144.71 (C-7), 174 (C-1). IR (KBr) *ν* (cm^−1^): 3301 (broad, N–H, O–H), 2923, 2852 (medium, C–H, aliphatic), 1384 (strong, N=N), 1727 (weak C=O), 1583 (strong, C=C), 1277 (strong, C–N),1075, 824. MS (EI, 70 eV): *m/z* (relative intensity) 411 (M^+^·, 3.8), 341 (7.69), 149 (66), 69 (100), 41 (55.5).

### 2S-RED: 2-((5-((2-hydroxynaphthalen-1-yl)diazenyl)-2,3-dimethylphenyl)amino)benzoic acid (C_25_H_20_N_3_O_3_)


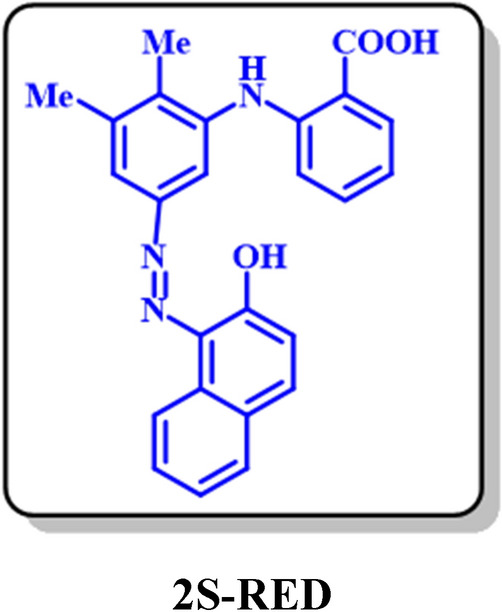


Isolated yield: 41%. Mp > 300 °C (Dec.). ^1^H NMR (500 MHz, CD_3_OD) *δ* (ppm): 2.23 (s, 3H, aliphatic), 2.34 (s, 3H, aliphatic), 6.89 (d, *J* = 8.9 Hz, 1H, aromatic), 7.03 (d, *J* = 7.3 Hz, 1H, aromatic), 7.12 (t, *J* = 8.0 Hz, 2H, aromatic), 7.21 (d, *J* = 8.1 Hz, 1H, aromatic), 7.4 (t, *J* = 8.5 Hz, 1H, aromatic), 7.58 (t, *J* = 8.0 Hz, 1H, aromatic), 7.78 (d, *J* = 8.0 Hz, 1H, aromatic), 7.81 (t, *J* = 8.0 3H, aromatic), 8.64 (d, *J* = 5.5 Hz, 1H, aromatic), 8.56 (s, 1H, aliphatic), 8.86 (d, *J* = 8.7 Hz, 1H, aromatic). ^13^C NMR (125 MHz, CD_3_OD): δ 12.83 (C-15), 19.22 (C-13), 113.1 (C-17), 119.9 (C-6), 121.51 (C-11), 121.9 (C-4), 123.9 (C-19), 125.52 (C-10), 125.99 (C-23), 127.08 (C-21), 127.66 (C-22), 128.4 (C-24), 133.41 (C-3), 137.81 (C-5), 150.31 (C-16) 139.91 (C-7), 138.73 (C-13), 132.83 (C-8), 131.29 (C-20), 126.92 (C-9), 125.6 (C-24), 123.8 (C-25), 123.6 (C-2), 169.1 (C-1). IR (KBr) ν (cm-1): 3301 (broad, N–H, O–H), 2923, 2852 (medium, C–H, aliphatic), 1727 (weak C=O), 1583 (strong, C=C), 1383 (strong, N=N), 1277 (strong, C-N), 1075, 824. MS (EI, 70 eV): *m/z* (relative intensity) 413 (M^+^·, 5.8), 368 (12), 239 (18), 149 (41), 43 (100).
